# LGBTQ+ Aging Research in Canada: A 30-Year Scoping Review of the Literature

**DOI:** 10.3390/geriatrics6020060

**Published:** 2021-06-12

**Authors:** Kimberley Wilson, Arne Stinchcombe, Sophie M. Regalado

**Affiliations:** 1 Department of Family Relations and Applied Nutrition, University of Guelph, Guelph, ON N1G 2W1, Canada; 2 Department of Recreation and Leisure Studies, Brock University, St. Catharines, ON L2S 3A1, Canada; astinchcombe@brocku.ca; 3 Northern Ontario School of Medicine, Thunder Bay, ON P7B 5E1, Canada; sregalado@nosm.ca

**Keywords:** aging, lesbian, gay, bisexual, transgender, queer, seniors

## Abstract

Canada has a unique socio-political history concerning the inclusion of lesbian, gay, bisexual, transgender, and queer (LGBTQ+) people. With aging populations, understanding diverse groups of older adults is paramount. We completed a systematic search and scoping review of research in Canada to quantify and articulate the scale and scope of research on LGBTQ+ aging. Our search identified over 4000 results and, after screening for relevance, our review focused on 70 articles. Five major themes in the literature on LGBTQ+ aging in Canada were identified: (1) risk, (2) HIV, (3) stigma, and discrimination as barriers to care, (4) navigating care and identity, (5) documenting the history and changing policy landscapes. Most of the articles were not focused on the aging, yet the findings are relevant when considering the lived experiences of current older adults within LGBTQ+ communities. Advancing the evidence on LGBTQ+ aging involves improving the quality of life and aging experiences for LGBTQ+ older adults through research.

## 1. Introduction

Gerontologists, health and social care providers, and decision-makers require evidence to develop theory and design policies and interventions to support aging individuals and aging populations. The discipline of gerontology is still relatively new, with much of the theory development occurring in the mid-twentieth century. In Canada, the gerontology community came together in 1971 under the national association, the Canadian Association on Gerontology (CAG). In the last fifty years, a wealth of evidence has been collected on aging; however, the field has been criticized for focusing narrowly on aging within dominant communities, e.g., [1,2]. To date, there remains a dearth of research focusing on diverse experiences of aging, e.g., [3,4,5] despite the known heterogeneity of aging individuals in Canada. 

Understanding and responding to the lived experiences of lesbian, gay, bisexual, transgender, and queer (LGBTQ+) older adults is particularly important given that this is the first generation to age ‘out’ (i.e., living openly regarding their sexual orientation and gender identity). One day before the Stonewall Riots in New York City, the decriminalization of homosexual acts between consenting adults received royal assent (27 June 1969) in Canada. Yet fifty years later, several provisions within the Criminal Code continue to criminalize queer sexual acts [6]. Influenced by the Stonewall riots of New York City in 1969, Canada’s first ‘gay rights’ march was held in Ottawa, Ontario, in 1971 [7]. Ten years later, after Toronto Police arrested just under 300 men in Canada’s own Stonewall, known as the Bathhouse Raids, the LGBTQ+ community engaged in mass protests [8]. 

Other important socio-historical contexts include the development of the ‘fruit machine’ between 1950 and the 1990s to facilitate the ‘gay purge’ [9] wherein federal employees suspected to be homosexual were subjected to testing and discharged from their roles [10]. With the first case of Acquired Immunodeficiency Syndrome (AIDS) identified in Canada in 1979, the AIDS crisis of the 1980s and 1990s had significant impacts on the LGBTQ+ communities, particularly gay men. Initially coined the “Gay-Related Immune Deficiency (GRID)” [11] (p. 9), HIV and AIDs added to the already pervasive stigma and discrimination against gay men and LGBTQ+ communities; it is important to consider the magnitude of losses for current older adults who lived through the crisis and the unique experiences of long-term survivors of the disease. 

In the following decades, incremental policy changes were implemented to improve equity between LGBTQ+ and majority populations, including the legalization of same-sex marriage in 2005, and the update to the Canadian Human Rights Act and the Criminal Code to include the terms “gender identity” and “gender expression”. Considering these landmark events along the life courses of current and future cohorts of aging LGBTQ+ individuals in Canada highlights the relevance of the minority stress model which posits that “circumstances in the environment, especially related to stigma and prejudice, may bring about stressors that LGBTQ+ people experience their entire lives” [12] (p. 209). 

Although there have been other reviews of the literature on LGBTQ+ aging, they have not had a Canadian focus, e.g., [13] and/or have not included the literature on infectious disease, e.g., [14], which is an important contribution to the history of literature on LGBTQ+ aging Canada. In order to understand the research gaps related to sexual and gender minority aging, we undertook a systematic search and scoping review of Canadian literature to quantify and articulate the scale and scope of research. Identifying the gaps in the literature is essential to move the state of science forward on LGBTQ+ aging in Canada. 

## 2. Methods

Scoping reviews are a useful technique to map the relevant research and understand the breadth of evidence within a particular field [15]. Arskey and O’Malley [15] propose four reasons why a scoping review may be conducted, including examining the “extent, range and nature of research activity” (p. 21) along with describing and disseminating the major finding of research and determining the gaps within the existing evidence base. Our goal was to examine the state of research on LGBTQ+ aging in Canada, to synthesize major themes in the research, and to identify gaps in research, making a scoping review an appropriate methodological choice. We followed Arskey and O’Malley’s [15] framework for conducting a scoping study wherein we identified our research question, identified studies relevant to our inclusion criteria, selected studies and charted the data that was extracted, and, finally, collated and summarized the major themes within the included research. 

At the outset of this study, our inclusion criteria were broad; as is common in scoping reviews, an iterative process allowed for the narrowing of our criteria through the study selection phase [4]. Our original inclusion criteria required that articles be written in English or French; focus at least in some part on lesbian, gay, bisexual, or transgender individuals; include participants aged 65 and older; and take place, at least in some part, in Canada. We also excluded any articles that did not describe empirical data (e.g., commentaries). Throughout our study selection, we narrowed our criteria to studies that included an overlap in our three major domains of interest (i.e., LGBTQ+, participants aged 65 and older, and data that were collected in Canada). 

A Health Sciences Librarian designed and executed all database searches with input from the other authors. Comprehensive and systematic searches were conducted in the following databases using a combination of subject headings and keywords: Ovid MEDLINE; Ovid EMBASE; Ovid PsycINFO; EBSCOhost CINAHL; and, Clarivate Web of Science, Core Collection. The search terminology included terms related to aging (e.g., aged, aging, etc.), gender/sexual minority identity (e.g., homosexual, bisexual, transgender, etc.) and Canada (e.g., Canada, Canadian, etc.). We initially developed the search in Ovid Medline using keywords and Medical Subject Headings (MeSH) and then translated these terms into each of the searched databases. Databases were searched 19 July 2017 with the exception of Clarivate Web of Science, Core Collection, which was searched on 20 July 2017. Restrictions were not imposed on language, study design, or date of publication; however, keywords and/or subject headings were used to limit to Canadian-driven results. The initial search results are summarized in Table 1. 

**Figure 1:**
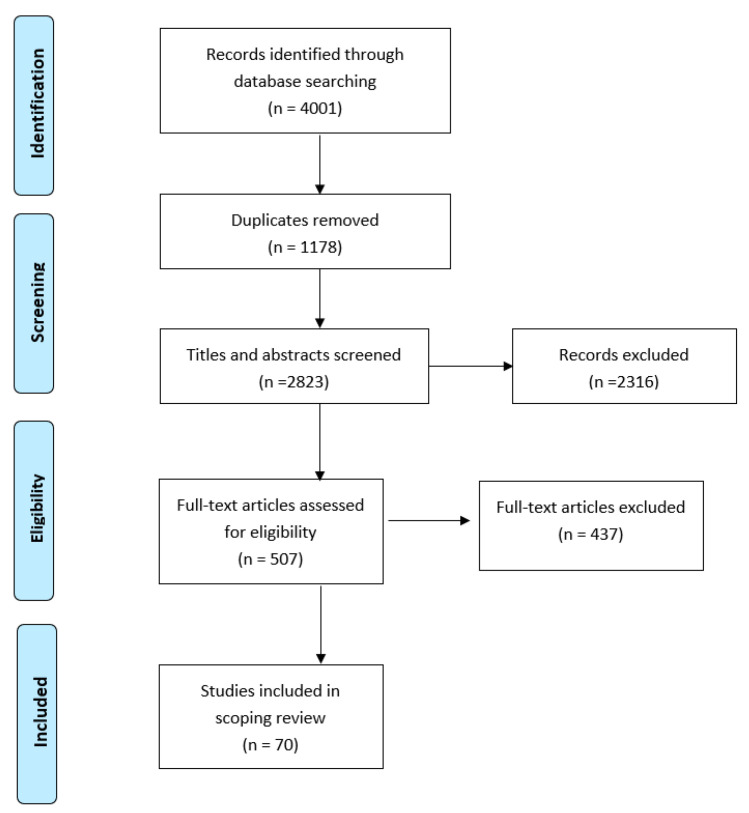
Flow diagram for scoping review showing literature search and selection.

Once duplicates were removed, two independent reviewers screened the titles, keywords, and abstracts of each 2823 records to determine if they met our inclusion criteria. After an independent review, they came together to confirm results; when there was disagreement, they reviewed the abstract together to reach consensus. After excluding 2316 articles, two independent reviewers assessed the full text of 507 papers to assess their eligibility for inclusion. At this stage, we also narrowed our focus to ensure that relevant articles met all three of our criteria simultaneously. This meant excluding articles that were not explicit about their sample. For example, in some articles, data were collected from heterosexual and sexual minority participants; each article was also reviewed to ensure that in addition to including data on LGBTQ+ individuals, they also needed to be over 65 and Canadian. After review, our final sample included 70 articles that met our inclusion criteria as summarized in Figure 1.

## 3. Results

Given our goal to review the full history of literature on LGBTQ+ aging in Canada, included articles ranged from 1986 to 2017 (the year of our search). There was significant methodological variation, with a fairly even mix of qualitive studies and quantitative studies, and few mixed methods studies. Few studies were longitudinal, the majority were cross-sectional. Methods of data collection included focus groups, chart reviews, observational, interviews, and surveys/questionnaires. A summary of the included articles can be found as Appendix A.

### 3.1. Major Themes in the Literature

After reading through each article, we identified five major themes in the literature on LGBTQ+ aging in Canada.

1. Risk: reducing it, predicting it, patterns of risk, and sociodemographic factors;
2. Improving HIV testing and the experiences of living with HIV;
3. Homophobia, transphobia, stigma, and discrimination as barriers to care;
4. Navigating care and identity;
5. Documenting history and changing policy landscapes.

### 3.2. Theme 1: Risk: Reducing It, Predicting It, Patterns of Risk, and Sociodemographic Factors

Among the 70 articles reviewed, 29 were focused on risk. We categorized risk in several ways: papers that focused on strategies and interventions to reduce risk, studies that sought to identify ways to predict risk, using evidence to find patterns of risk, and linking risk to certain sociodemographic factors. Many of the papers were interrelated in their focus on risk, and often there were intersections with the second theme, with much of the literature on risk also focusing on HIV and AIDS. Areas of risk that were common in the literature included: infectious diseases (e.g., HPV infections co-occurring among HIV-infected men [16]), drug-use amongst sexual minority participants [17] bacterial and viral sexually transmitted infections [18] and “risky behaviours” [19].

The temporal aspect of research in this domain likely influenced the wave of research on risk and risk reduction, aligning with the HIV/AIDS crisis. For example, Soskolne, Coates, and Sears [20] published an early study looking at the characteristics of the homosexual and bisexual population in Toronto, Ontario. Moreover, Chandarana, Conolon, Noh, and Field [21] investigated the relationship between worry and concern about AIDS to perception of risk, knowledge of AIDS, change in sexual behaviour, feelings of control over health, and sociodemographic factors among 148 gay men, finding that approximately 70% of participants were worried about the possibility of contracting an HIV infection. Ferro and Salit [22] aimed to describe the clinical and epidemiological characteristics on HIV infections among patients aged 55 and older while comparing them with younger participants. Based on data from all cases of HIV presented to an outpatient clinic in Toronto between May 1984 and May 1990, researchers found that during the study period 67% of the older participants had died and only two from non-AIDS related sources. 

Many studies focused on documenting infectious disease exclusively within samples of men who have sex with men (MSM), including human papillomavirus (HPV) infections [16], Ciproflaxacin-resistant Shigella sonnei [23], syphilis [24], and hepatitis B [25]. As a result, intervention research that focused on reducing risk aligned; studies explored the acceptability of HPV vaccination [26] and the effectiveness of a hepatitis B vaccination program [27]. Earlier literature focused on AIDS reduction educational interventions with gay and bisexual men [28]. 

Similarly, articles that focused on ‘risky’ or ‘at-risk’ behaviours focused on men, e.g., [29,30]. Myers and colleagues [31] examined ‘unsafe’ sex practices among gay and bisexual men. Their results indicated that the rates of unprotected anal intercourse were significantly higher within long-term partnerships whereas those who had more casual sex tended to have safer sex practices. Cox and colleagues [19] examined data from 364 MSM to assess the impact of antiretroviral treatments on perceptions of ‘at-risk’ sexual behaviours of HIV positive MSM. Within this study ‘at-risk’ behaviour was focused on penetrative sex without a condom at least one time in the last six months. In total, 34% of participants reported at-risk behaviours; participants perceived reduced safe sex practices due to the introduction of HIV treatments and perceived lower HIV transmission risk when taking anti-retroviral treatments. Shuper and Fisher [32] examined the role of sexual arousal on sexual decision-making processes and ‘risky sex’ intentions of HIV positive MSM, finding that sexual arousal impacted participants decision or intention to engage in risky sexual behaviours (specifically receptive anal intercourse). 

The literature within this thematic area began to develop a socio-demographic risk profile, including health behaviours such as smoking, e.g., [33], body mass index, and drive for masculinity [34]. Hogg, Craib, Willoughby, Sestak, Montaner, and Schechter [35] sought to understand if there were sociodemographic predictors of high-risk HIV behaviours in seronegative gay men. Within this study, participants were deemed ‘risk takers’ if they reported unprotected anal intercourse with casual partners. They found that age (younger), education (less educated), income (low income), heavy drinking, smoking, and use of nitrite inhalants (i.e., poppers) increased risky behaviour in HIV seronegative gay men [35]. Adding to the sociodemographic profile of risk, Lessard, Lebouché, Engler, and Thomas [36] compared immigrant and non-immigrant men visiting an HIV testing site. They found that immigrant clients had a lower income and more education and were younger and more often unemployed. Fewer immigrant men reported ‘risky’ sexual behaviours such as unprotected sex with a HIV-positive partner or receiving goods or services in exchange for sex [36]. Ross and colleagues [37] have contributed to the literature on bisexuality with a specific focus on mental health and substance use and the relationship between bisexuality, low socio-economic status, and mental health [38]. These studies also contribute to a ‘sociodemographic risk profile’ that moves beyond a focus on MSM. 

The sociodemographic profiles of risk also help to offer variables that protect against risk. For example, Moody and Smith [39] looked at suicide protective factors among transgender adults, finding that social support from family and friends and optimism were significantly and negatively correlated with suicidal behaviour. In Chandarana and colleagues [21] research, they found that there was a significant relationship between worry and concerns about AIDS and reduction in risk behaviours of participants in their research. Findings from the Omega Cohort study indicated that men in their study were more likely to receive vaccination against the hepatitis B virus (HBV) if they were having sex in bathhouses and if they had consulted with a physician about their sexual orientation in the previous six months [40].

Within the literature on risk, only a minority of studies examined women, e.g., [41]. In addition to Ross and colleagues’ work with bisexual individuals, Persson, Pfaus, and Ryder [42] explored mental health disparities in non-monosexual women. They found that when compared to monosexual women, non-monosexual women were more likely to have experienced childhood abuse, had higher rates of risky sexual behaviour, and reported less disclosure of sexual identity. The authors posited risky sexual behaviours and identity concealment explain the increased depressive symptoms and anxiety amongst this population. Ploeg, Lohfeld, and Walsh [43] conducted focus groups with a variety of marginalized older adults and identified lesbian women as such a group. Within this study, participants were asked specifically about elder abuse; lesbian women perceived that they were at higher risk for isolation and emotional abuse [43].

Research stemming from the Trans Pulse Project offers insight into sexual risk among transgender people in Ontario. Within their sample of 433 participants, three per cent were over the age of 65. Bauer, Travers, Scanlon, and Coleman [44] identified the most common high-risk behaviours among female-to-male (FTM) and male-to-female (MTF) participants within the sample including unprotected receptive genital sex and insertive genital sex. Scheim, Bauer, and Shokoohi [45] explored disparities and predictors of heavy episodic drinking of participants within the Trans Pulse Project. They found that roughly one third of participants reported heavy episodic drinking at least monthly in the past year and 10.9% reported weekly or more. 

Using Trans PULSE data, Rotondi and colleagues [46] examined nonprescribed hormone use and self-performed surgeries. Only 6.4% of the sample were using non-prescribed hormones; however, they face critical risks pertaining to dosing and a lack of monitoring. Further, as the authors note, the decision to perform surgery on oneself creates significant risk. These data offer unique contributions to the theme of risk and stand in contrast to bulk of the literature within this theme.

### 3.3. Theme 2: Improving HIV Testing and the Experiences of Living with HIV

Although related to Theme 1, which focused on risk and infectious disease risk, we identified a second theme that pertained specifically to improving HIV testing and the experience of living with HIV/AIDS. In the early wave of research, the focus was often on exploring the intentions of gay and bisexual men to take the HIV antibody test, e.g., [47] as well as the influence of various factors such as socio-demographic, lifestyle, and geographic factors on test taking and sexual behaviour, e.g., [48]. Myers, Orr, Locker, and Jackson [49] collected data from over 1200 gay and bisexual men in Toronto to identify factors that impacted their decision to be tested for HIV. Respondents emphasized the need for anonymity and noted that geography influenced safer-sex practices. In more recent literature, Gilbert and colleagues [50] studied the acceptability of internet-based of HIV and STI testing for MSM. Amongst their sample of 8388 men participants ranged in age from 13–84 years old; however, they found that the acceptability was higher for those who were younger than age 30. 

Much of the following research continued to be conducted in urban centres (namely Montreal, Toronto, and Vancouver) and nearly exclusively focused on MSM. With a strong health promotion framing, researchers evaluated interventions that improved referrals and reduced time between diagnosis and treatment for gay and bisexual men [51], including targeted rapid testing sites for immigrant MSM [52]. Focusing on prevention of infectious disease, Murry and Adam [53] conducted interviews with gay men in Toronto to understand the age-related components of safer-sex practices. Based on their findings the authors suggest that one of the most effective strategies to prevent HIV transmission is to create communities of support within the gay community. 

Similar to the socio-demographic profiles associated with risk, Low-Beer et al. [54], conducted telephone interviews with individuals living in the West End of Vancouver. From their sample of 1176 they identified 300 as gay or bisexual and identified a 16% prevalence rate of HIV-positive within their sample. They noted income and employment gaps between the HIV positive men and the rest of the sample, with HIV-positive gay and bisexual men more likely to have an annual income of under 20,000 CAD. Brennan, Emlet, Brennensthul, and Rueda [55] used data from the Ontario HIV Treatment Network Cohort study to present the socio-demographic characteristics of HIV positive adults, focusing on those aged 50 and older. While they presented evidence of disparities between sexual minority and majority participants, they also moved away from solely focusing on a risk profile and highlighted that among the oldest participants, 80% reported that their health was either excellent or good. 

In addition to improving testing of HIV, literature within this thematic area also examined the experience of living with HIV. This included research on the physical impacts, such as gastrointestinal symptom distress, e.g., [56], and mental health impacts, e.g., [57]. The impact of stigma was also highlighted, including the intersection of the stigma of HIV with other lived experiences and social locations. Furlotte and Schwartz (57) interviewed 11 participants (nine men, two women) aged 52–67. Themes of uncertainty, stigma and resilience were identified within these data and in particular the recognition that aging can compound existing stigmas, such as the stigma surrounding HIV. Liboro and Walsh [58] interviewed nine HIV-positive gay Catholic men, anticipating that their religious affiliation would have exacerbated the stigmas they endured related to their HIV status and sexual orientation; however, they found that faith was identified as source of strength and support for participants.

### 3.4. Theme 3: Homophobia, Transphobia, Stigma, and Discrimination as Barriers to Care

Much of the research identified in this theme was focused on stigma and discrimination, particularly homophobia and transphobia, and the barriers these created when accessing care. The literature was focused on younger participants but included some older adults, e.g., [59,60]. The results of all these studies speak to the frequency that individuals face stigma and discrimination when accessing care. Morrison [61] investigated the frequency of perceived discrimination experienced by a sample of gay men and lesbian women and the linkage to psychological well-being. Although they found that 80% of participants (*n* = 278) reported having an experience of being verbally insulted due to sexual orientation, overall participants were not psychologically distressed. Morrison also found that on average, gay men who had experiences with discrimination also showed evidence of greater depression and through their analyses demonstrated an inverse relationship between the greater experiences of discrimination and life optimism and self-esteem. Recognizing this frequency Logie and Earnshaw [62] reported on the psychometrics of a multi-dimensional sexual stigma scale to measure both perceived and enacted stigma for lesbian, bisexual and queer women, a tool that could be used for future measurement in healthcare settings. 

Literature on stigma and discrimination focused on screening and prevention [59,60] and mental health and recovery [63]. Across studies, there were consistencies in the gaps in services received by sexual and gender minority participants. Giblon and Bauer [64] examined the experiences of transgender and cisgender residents of Ontario, finding that the rate of unmet needs was far greater for transgender people. 

Brotman and colleagues [65] specifically looked at the experiences of caregivers of gay and lesbian older adults in Canada. The results from their research suggest that the recipients of care experience subtle forms of discrimination from health care professionals and expressed fear about being discriminated about in future health care interactions. This fear may result in reducing utilization and access to health care services which downloads responsibility onto informal carers and care networks. Although only two of the 17 participants in the study by Brotman et al. [65] were over 65 years old, this article offers important insights and considerations for the aging population in Canada. 

### 3.5. Theme 4: Navigating Care and Identity

While there are overlaps in thematic areas, the concept of navigating care and identity was strongly identified in many of the articles that focused on health or social services. Many of the challenges associated with navigating care and identity stem from the hetero- and cis-normative assumptions that are embedded within care interactions [66] and negative experiences within the health care system, e.g., [67]. Within this theme, we identified three content areas: aging, cancer care, and mental health services. 

Although many of the articles in this theme included some older participants, there was a cluster of research within this theme that focused on aging. In particular the experience of older lesbians and bisexual women’s experience of home care settings [68,69,70], older lesbian and gay couples and their hopes and fears around home care and long-term care services [71]. Chamberland [72] interviewed lesbian women aged 60–76 and found that they face issues such as economic insecurity, isolation and have fears around identity disclosure. Participants in Chamberland’s [72] research indicated a preference for an all lesbian, all woman, or all gay/lesbian residence as they grow older. These interviews were contrasted with service providers who acknowledged having little knowledge about the life experiences and psychosocial needs of lesbian women. Across the studies focused on aging, fears about identity disclosure, discrimination, and nuanced care were woven throughout; as Grigorovich [70] noted, service providers need to be both technically and emotionally competent in their work given the intimacy of caring and providing services within the home of an LGBTQ+ person. 

The experience of LGBTQ+ persons navigating cancer care was also highlighted in several articles within this theme. Sinding, Barnoff, and Grassau [73] examined homophobia and heterosexism in cancer care for lesbian women in Ontario. The majority of participants within their study had not experienced any kind of discrimination; however, they made an effort to avoid homophobia by not coming out to their providers. Participants suggested that positive cancer care was the absence of homophobia, and negative cancer resulted in being denied standard care, ignoring aspects of lesbian identity that were directly related to cancer care, and not having lesbian-positive support. The lack of support groups for gay and lesbian cancer patients was echoed by Katz [74]. Katz [74] examined the experience of gay and lesbian patients with cancer. Here too, the focus on disclosure was exacerbated by the diagnosis and treatment challenges. The researcher also noted that the recognition of one’s partner by the care team and in particular the physician’s response to the partner’s presence was a component of the recovery process.

Finally, Eady, Dobinson, and Ross [75] examined bisexual people’s experiences with mental health services. In particular, they looked at the perceptions of providers’ feelings towards their sexuality and whether they believed their needs were being met. Participants were able to identify both negative and positive experiences; however, overall, the majority of participants experiences with mental health providers were categorized as negative.

### 3.6. Theme 5: Documenting History and Changing Policy Landscapes

A theme we identified in the more recent literature reflected shifts in the policy landscape that allow for same-sex marriage in Canada. Alderson [76] conducted a phenomenological study interviewing same sex couples in three countries, including Canada, to understand the experience of those who had married already. They found that those in same-sex marriages found power in the institution of marriage and felt empowered in their marriages but held internalized stigma and struggled with shame and fear. Following the introduction of the Civil Marriage Act in 2005 in Canada, MacIntosh, Reissing, and Andruff [77] built on Alderson’s seminal study and conducted research to assess the impact of marriage with the first cohort of those who had legally same-sex marriages in Canada. Using both surveys and interviews, they found that participants in their study had higher levels of relationship satisfaction and lower attachment-related anxiety and avoidance when comparing means with heterosexual couples in other research. Further, through their thematic analysis, they identified that participants had an overall positive experience of marriage in social, relation, and political elements. Humble [78] specifically looked at the experience of older same-sex couples who married, interviewing 28 mid to late-life lesbian, gay, and bisexual (LGB) individuals who married after the change to Canadian policies. Participants explained why and how they got married and the researcher identified two unique stages through their analysis, integration, and intentionality. Participants described the complexities of incorporating the idea of marriage into something that a LGB person can do into their psyche and deliberately planning part of their wedding to include features related to their sexual orientation. Similar to Humble’s [78] findings, Lyon and Frohard-Dourlent [79] explored the experience of same-sex common law partners; in their study, the authors found that for those who opted not to marry did so in in part due to the fact that it was previously illegal and inaccessible to them and it was still associated with heterosexuality.

In addition to the recent literature on changing policy landscapes, through this scoping review, we identified some of the earliest writing on older gay men in Canada, a first step in acknowledging their presence in the academic literature. In 1989, Lee [80] published in the Canadian Journal on Aging results of two studies. The first was a qualitative study containing two groups of elderly homosexual men. Data were collected through attendance counts, participant observation, informal interviews, and note taking of individuals attending gay two gay organizations, CODA (Came Out Decades Ago), and GALA (Gays and Lesbians Aging) events. The second was a four-year longitudinal study of men (*n* = 47) spanning from 50–80 years of age (*M* = 60.5). These studies investigated the cultural differences that exist between young and old gay men. Results of these studies suggested that there was no significant relationship between ‘coming out’ and life satisfaction among older gay men. However, there was a relationship noted between participating in the gay community and life satisfaction, such that those who reported that they were happy were more likely to be a part of gay community organizations. ‘Coming out’ experiences continued to be documented over time; Hoffarth and Bogaert [81] investigated psychosocial correlates, including personality factors, of ‘coming out’ experiences among gay and bisexual men.

## 4. Discussion

The purpose of this scoping review was to quantify and articulate the scale and scope of research on LGBTQ+ aging in Canada over a 30-year period. Our goal was to map themes within the literature and identify gaps to move the state of science forward on LGBTQ+ aging in Canada. After screening for relevance, our team identified 70 studies that met our search criteria. Through analysis of the literature, we identified five themes.

The majority of the literature was focused on narratives of risk: how to reduce it, predicting those at risk, and sociodemographic contribution factors. Although separated out as a second theme, the focus on HIV testing and living with HIV was primarily articulated from a risk narrative. Gay men and men who have sex with men (MSM) were almost exclusively the population of interest in studies about risk and HIV. Over a 30-year period, the dominant narrative in the Canadian research literature that included older adults labelled gay men and MSM’s sexual activities and practices as ‘risky’. In doing so, while contributing to the literature around interventions to reduce risk, there may also be the unintended effect of perpetuating pathology and stigma. Further, this focus on ‘risk’ and infectious disease excludes the experience of those within LGBTQ+ communities who are not men. The diversity of the LGBTQ+ communities is underrecognized within this body of literature. Further, very few of these articles were focused on if and how aging impacts risk; however, given that at least one participant in the sample met our inclusion criteria, they were accounted for within our search. In fact, the majority of these articles had no focus on age as a sociodemographic variable of interest. The articles on risk were some of the earliest in terms of the chronology of research on LGBTQ+ aging in Canada. From a life course perspective, it is important to note this historical context. For those who are now older adults, their lifetime of research experiences may have been limited to studies focusing on infectious disease and with unintended effects of stigmatizing sexual behaviours. When considering working with older adults, particularly older men who have sex with men, it is necessary to recognize that building trust and credibility may be an important part of designing research and engaging participants in the research process.

The history of research on LGBTQ+ aging in Canada illustrates clearly how homophobia, transphobia, stigma, and discrimination serve as barriers to care and full participation in society. When working with older adults from LGBTQ+ communities, it is important to recognize how ageism intersects with these other forms of stigma and discrimination, particularly as individuals grow older and are faced with navigating care and determining safety around their identity. Meyer’s [82] minority stress model highlights how LGBTQ+ individuals face additional internal and external stressors related to their minority status, including discrimination, identity concealment, and increased vigilance. This maps onto much of the literature under themes three and four, wherein the literature focuses on stigma, discrimination, and the internal process of navigating accessing care.

The fifth theme identified within the literature was centred around the impact of changing policies related to sexual orientation in Canada. The research within this theme underscores the significance of social and historical contexts on an individual’s trajectory. With a gerontological lens, it is important to note that most of the current older adults within LGBTQ+ communities in Canada grew up under discriminatory policy legacies, and these must not be forgotten.

Taken together, the history of research on LGBTQ+ aging in Canada is mostly focused on infectious disease, namely HIV, and on reducing the risk for men who have sex with men. In fact, although the papers included met the criteria of including participants over the age of 65, very little of the research within this review is actually focused on aging or makes recommendations about the impact on aging individuals, with few notable exceptions, e.g., [68,69,70,71,72,78,83]. Those that focused on aging as a key element of their research tended to focus on the impact of stigma and discrimination and navigating care and identity, themes that are especially salient for those who may have changing health and social care needs as they grow older.

This paper offers an important contribution to the literature on LGBTQ+ aging in Canada. It highlights the evidence base over a 30-year period and identifies key themes in the literature. This research is not without limitations. As an example, many studies we reviewed in our screening process did not include the range of ages of participants and thus were excluded. In the future, researchers are encouraged to be explicit about the range of ages of participants in their studies, even if the focus is on aging. We also note that while our intention was to focus on aging, much of the literature we reviewed was not actually about aging experiences. Further, since the systematic search for this scoping review was conducted, there have been some important contributions to the Canadian literature that have helped to move the field along, e.g., [84,85,86].

Through the process of mapping the literature and identifying themes, our team also identified gaps within the research. The strong focus on risk and infectious disease also highlights the lack of strengths-based research and focus on resilience. The ‘crisis competence’ model suggests that LGBTQ+ individuals may have methods to draw upon from their experiences navigating and adapting to stigma early in life that can be useful when facing age-related changes [13]. This model offers important considerations for future research.

Recognizing the social and historical contexts and possible experiences with researchers throughout their life courses, older adults should be included in the design of future research where possible, and research teams should be prepared to invest time in building trust and credibility with participants. There remains relatively little research on the experience of aging for trans folks and for individuals with intersecting identities (e.g., Black, Indigenous, and people of colour). To have evidence informed policy and practice, we need a stronger evidence base to respond to the needs of an aging LGBTQ+ population.

The bulk of the literature included in this review was not focused on the aging process, yet the findings of this review are relevant when considering the lived experiences of current older adults within LGBTQ+ communities. The studies within this review that focused on aging were largely descriptive in nature.

## 5. Conclusions

The next wave of research on LGBTQ+ aging in Canada should move beyond identifying needs and into addressing needs through intervention. Despite the importance of the local context, there are opportunities to leverage findings from research and best practices in other jurisdictions. Much of the research in this synthesis proposed avenues for future research that included educational interventions for health and social care providers. Participants in the research suggested these interventions would reduce their fears and may serve to reduce stigma and discrimination. With the world’s growing population of older adults, there is an imperative to move research into action to improve the quality of life and aging experiences for LGBTQ+ older adults. While these findings are specific to Canada, they may provide insights into the experiences of LGBTQ+ aging in other jurisdictions.

## Figures and Tables

**Table 1:** Initial systematic search results by database.

Database	Initial Results
Medline	1426
PsycINFO	430
Embase	1073
Web of Science	820
CINAHL	252
TOTAL	4001

## Data Availability

Data sharing not applicable.

## Appendix A

**Table A1:** Summary of included articles [87,88,89].

Citation	Study Information
Alderson, K. (2004). A phenomenological investigation of same-sex marriage. *The Canadian Journal of Human Sexuality*,*13*(2), 107–122.	Age: Range = 30–66; mean = 46.3 65+: 1 Gender: M and F LGBTQ+ Inclusion: Gay and lesbian same-sex couples Sample Size: 43 Location: International Approach: Qualitative
Bauer, G. R., Travers, R., Scanlon, K., & Coleman, T. A. (2012). High heterogeneity of HIV-related sexual risk among transgender people in Ontario, Canada: A province-wide respondent-driven sampling survey. *BMC Public Health*, *12*(1), 292.	Age: Range = 16–65+ 65+: 3% Gender: M and F LGBTQ+ Inclusion: FTM, MTF Sample Size: 433 Location: Ontario Approach: Quantitative
Bergeron, S., & Senn, C. (2003). Health care utilization in a sample of Canadian lesbian women: Predictors of risk and resilience. *Women & Health*, *37*(3), 19–35, doi:10.1300/J013v37n03_02	Age: Range = 18–67; mean = 38.85 Gender: F LGTBQ+ Inclusion: Lesbian women Sample Size: 254 Location: Canada Approach: Quantitative
Bossio, J. A., Pukall, C. F., & Bartley, K. (2015). You either have it or you don’t: The impact of male circumcision status on sexual partners. *The Canadian Journal of Human Sexuality*, *24*(2), 104–119.	Age: M: Range = 20–71; mean = 31.50, F: Range = 19–57, mean = 26.87 Gender: M and F LGBTQ+ Inclusion: Gay men Sample Size: 196 Location: International Approach: Quantitative
Brennan, D., Craig, S., & Thompson, D. (2012). Factors associated with a drive for muscularity among gay and bisexual men. *Culture, Health & Sexuality*, *14*(1), 1–15, doi:10.1080/13691058.2011.619578.	Age: Range =16–76; mean = 34.1 Gender: M LGBTQ+ Inclusion: Gay and bisexual men and other men who have sex with men Sample Size: 400 Location: Toronto, Ontario Approach: Quantitative
Brennan, D. J., Emlet, C. A., Brennenstuhl, S., & Rueda, S. (2013). Socio-demographic profile of older adults with HIV/AIDS: Gender and sexual orientation differences. *Canadian Journal on Aging/La Revue Canadienne du Vieillissement*, *32*(1), 31–43.	Age: Range = 50–86; mean = 57, SD = 6.2 65+: 136 Gender: M and F LGBTQ+ Inclusion: Gay and Bisexual Men Sample Size: 1103 Location: Ontario Approach: Quantitative
Brotman, S., Ryan, B., Collins, S., Chamberland, L., Cormier, R., Julien, D., Meyer, E., Peterkin, A., & Richard, B. (2007). Coming out to care: Caregivers of gay and lesbian seniors in Canada. *The Gerontologist*, *47*(4), 490–503, doi:10.1093/geront/47.4.490	Age: Range = 36–72; mean = N/A 65+: 2 Gender: M and F LGBTQ+ Inclusion: Gay men and lesbian women Sample Size: 17 Location: Montreal, Qubece; Halifax, Nova Scotia; and Vancouver, British Colubmia Approach: Qualitative
Brownrigg, B., Taylor, D., Phan, F., Sandstra, I., Stimpson, R., Barrios, R., ... & Ogilvie, G. (2017). Improving linkage to HIV care at low-threshold STI/HIV testing sites: An evaluation of the Immediate Staging Pilot Project in Vancouver, British Columbia. *Canadian Journal of Public Health*, *108*(1), e79–e84.	Age: Range = 19–71; mean = 32.3 Gender: M LGBTQ+ Inclusion: Men who have either male or female sexual partners Sample Size: 108 Location: Vancouver, British Columbia Approach: Quantitative
Chamberland, L. (2003). Elderly women, invisible lesbians. *Canadian Journal of Community Mental Health= Revue Canadienne de Sante Mentale Communautaire*, *22*(2), 85–103.	Age: Range = 60–76 Gender: F LGBTQ+ Inclusion: Lesbian women Sample Size: 20 Location: Montreal, Quebec Approach: Qualitative
Chandarana, P. C., Conlon, P., Noh, S., & Field, V. A. (1990). The AIDS dilemma: Worry and concern over AIDS. *Canadian Journal of Public Health= Revue Canadienne de Sante Publique*, *81*(3), 222–225.	Age: Range = 19–66; mean = 34 Gender: M LGBTQ+ Inclusion: Homosexual and bisexual men Sample Size: 148 Location: Canada Approach: Qualitative
Chow, C., Vallance, K., Stockwell, T., Macdonald, S., Martin, G., Ivsins, A., ... & Duff, C. (2013). Sexual identity and drug use harm among high-risk, active substance users. *Culture, health & sexuality*, *15*(3), 311–326.	Age: Range = 14–76; mean = 30.5 Gender: M, F, Transgender LGBTQ+ Inclusion: Gay, lesbian, bisexual, transgender individuals Sample Size: 2353 Location: Vancouver and Victoria, British Columbia Approach: Qualitative
Clarke, M. P., & Coughlin, J. R. (2012). Prevalence of smoking among the lesbian, gay, bisexual, transsexual, transgender and queer (LGBTTQ) subpopulations in Toronto—The Toronto Rainbow Tobacco Survey (TRTS). *Canadian journal of public health*, *103*(2), 132–136.	Age: Range =13–91 mean = 35.3 Gender: M, F and Transgender LGBTQ+ Inclusion: lesbian, gay, bisexual, transsexual, transgender and queer individuals Sample Size: 3,140 Location: Toronto, Ontario Approach: Quantitative
Colpitts, E., & Gahagan, J. (2016). “I feel like I am surviving the health care system”: understanding LGBTQ health in Nova Scotia, Canada. *BMC Public Health*, *16*(1), 1005–1012, doi:10.1186/s12889-016-3675-8.	Age: Range = mid 20’s–late 60’s Gender: Not reported LGBTQ+ Inclusion: LGBTQ individuals Sample Size: 20 Location: Truro and Halifax, Nova Scotia Approach: Qualitative
Cox, J., Beauchemin, J., & Allard, R. (2004). HIV status of sexual partners is more important than antiretroviral treatment related perceptions for risk taking by HIV positive MSM in Montreal, Canada. *Sexually Transmitted Infections*, *80*(6), 518–523, doi:10.1136/sti.2004.011288.	Age: Range = 24–73; mean = 45 Gender: M LGBTQ+ Inclusion: Men who have sex with men Sample Size: 346 Location: Montreal, Quebec Approach: Quantitative
Das, A. (2012). LGBTQ Women and Mental Health “Recovery.” *Psychiatric Rehabilitation Journal*, *35*(6), 474–475, doi:10.1037/h0094583.	Age: Range = 24–66; mean = N/A Gender: F LGBTQ+ Inclusion: LGBTQ women Sample Size: 13 Location: Southern Ontario Approach: Qualitative
Deonarine, A., Ogilvie, G., Montgomery, C., Makaroff, S., Holgerson, N., Grennan, T., ... & Wong, J. (2016). Trends in Syphilis partner notification among gay, bisexual, and other men who have sex with men in British Columbia, 2010 to 2013. *Sexually Transmitted Diseases*, *43*(8), 489–493.	Age: All years; mean = 42; SD = 12 years 70+: 5 Gender: M LGBTQ+ Inclusion: Men who have sex with men Sample Size: 350 Location: British Columbia Approach: Quantitative
de Pokomandy, A., Rouleau, D., Ghattas, G., Vézina, S., Coté, P., Macleod, J., Allaire, G., Franco, E., Coutlée, F., Allaire, G., Baril, J., Boissonnault, M., Charest, L., Charron, M., Coté, P., Coté, S., Coutlée, F., de Pokomandy, A., Dion, H., … Franco, E. (2009). Prevalence, clearance, and incidence of anal human papillomavirus infection in HIV-infected men: the HIPVIRG cohort study. *The Journal of Infectious Diseases*, *199*(7), 965–973, doi:10.1086/597207	Age: Range = 21–66; mean = 43 60+: 5 Gender: M LGBTQ+ Inclusion: Men who have sex with men Sample Size: 247 Location: Montreal, Quebec Approach: Quantitative
Dufour, A., Alary, M., Otis, J., Noël, R., Remis, R. S., Mâsse, B., ... & Vincelette, J. (2000). Correlates of risky behaviors among young and older men having sexual relations with men in Montreal, Quebec, Canada. *JAIDS-HAGERSTOWN MD-*, *23*(3), 272–278.	Age: Range = 16–73; mean = 33 Gender: M LGBTQ+ Inclusion: Men who have sex with men Sample Size: 810 Location: Montreal, Quebec Approach: Quantitative
Dufour, A., Alary, M., Otis, J., Remis, R. S., Mâsse, B., Turmel, B., ... & Omega Study Group. (2000). Risk behaviours and HIV infection among men having sexual relations with men: Baseline characteristics of participants in the Omega Cohort Study, Montreal, Quebec, Canada. *Canadian Journal of Public Health*, *91*(5), 345–349.	Age: Range = 16–73; mean = 33 Gender: M LGBTQ+ Inclusion: Men who have sex with men Sample Size: 810 Location: Montreal, Quebec Approach: Quantitative
Dufour, A., Remis, R. S., Alary, M., Otis, J., Mâsse, B., Turmel, B., ... & Omega Study Group. (1999). Factors associated with hepatitis B vaccination among men having sexual relations with men in Montreal, Quebec, Canada. *Sexually Transmitted Diseases*, *26*(6), 317-324.	Age: Range = 16–73; mean = 34 Gender: M LGBTQ+ Inclusion: Men who have affective and sexual relations with men Sample Size: 653 Location: Montreal, Quebec Approach: Quantitative
Eady, A., Dobinson, C., & Ross, L. E. (2011). Bisexual people’s experiences with mental health services: A qualitative investigation. *Community Mental Health Journal*, *47*(4), 378–389.	Age: Range = 16–69 60+: 2 Gender: M, F, Transgender LGBTQ+ Inclusion: Bisexual male, female and transgender individuals Sample Size: 55 Location: Ontario Approach: Qualitative
Ferro, S., & Salit, I. E. (1992). HIV infection in patients over 55 years of age. *Journal of Acquired Immune Deficiency Syndromes*, *5*(4), 348–353.	Age: (1) Range = 55–72; mean = 60.1; (2) Range = 21-39; mean = 30.1 Gender: LGBTQ+ Inclusion: HIV Positive Individuals Sample Size: (1) N = 33, (2) N = 58 Location: Toronto, Ontario Approach: Quantitative
Furlotte, C., Gladstone, J. W., Cosby, R. F., & Fitzgerald, K. (2016). “Could we hold hands?”: Older lesbian and gay couples. *Canadian Journal on Aging, 35*(4), 432–446.	Age: Range = 39–75, mean = N/A Gender: M and F LGBTQ+ Inclusion: Same-sex couples Sample Size: 24 Location: Canada Approach: Quantitative
Furlotte, C., & Schwartz, K. (2017). Mental health experiences of older adults living with HIV: Uncertainty, stigma, and approaches to resilience. *Canadian Journal on Aging/La Revue canadienne du vieillissement*, *36*(2), 125–140.	Age: Range = 52–67; mean = 60 Gender: M and F LGBTQ+ Inclusion: Gay, bisexual and two-spirited individuals Sample Size: 11 Location: Ottawa, Ontario Approach: Qualitative
Gaudreau, C., Ratnayake, R., Pilon, P., Gagnon, S., Roger, M., & Levesque, S. (2011). Ciprofloxacin-resistant Shigella sonnei among men who have sex with men, Canada, 2010. *Emerging Infectious Diseases*, *17*(9), 1747–1750, doi:10.3201/eid1709.102034	Age: M: Range = 20–65; mean = 40, F: Range = 45–50; mean = N/A 65+: 1 Gender: M and F LGBTQ+ Inclusion: Men who have sex with men Sample Size: 13 Location: Montreal, Quebec Approach: Mixed-methods
Giblon, R., & Bauer, G. (2017). Health care availability, quality, and unmet need: a comparison of transgender and cisgender residents of Ontario, Canada. *BMC Health Services Research*, *17*(1), 283, doi:10.1186/s12913-017-2226-z.	Age: 16+; mean = N/A 65+: 2.5% Gender: M, F, Transgender LGBTQ+ Inclusion: Transgender men and women Sample Size: 433 Location: Ontario Approach: Quantitative
Gilbert, M., Hottes, T. S., Kerr, T., Taylor, D., Fairley, C. K., Lester, R., ... & Ogilvie, G. (2013). Factors associated with intention to use internet-based testing for sexually transmitted infections among men who have sex with men. *Journal of Medical Internet Research*, *15*(11), e254.	Age: Range = 13–84; mean = 30.1 Gender: M LGBTQ+ Inclusion: Gay and bisexual men Sample Size: 8388 Location: Canada Approach: Qualitative
Godin, G., Myers, T., Lambert, J., Calzavara, L., & Locker, D. (1997). Understanding the intention of gay and bisexual men to take the HIV antibody test. *AIDS education and prevention: official publication of the International Society for AIDS Education, 9*(1), 31–41.	Age: Range = 16–75 mean = 32.7 Gender: M LGBTQ+ Inclusion: gay and bi-sexual men Sample Size: (Total sample)= 4803 (Included Sample)= 1512 Location: Canada Approach: Quantitative
Grigorovich, A. (2015a). Negotiating sexuality in home care settings: older lesbians and bisexual women’s experiences. *Culture, Health & Sexuality*, *17*(8), 947–961, doi:10.1080/13691058.2015.1011237	Age: Range = 55–72; mean = 63.9 Gender: F LGBTQ+ Inclusion: Women who identify as lesbian, bisexual or related term Sample Size: 16 Location: Ontario Approach: Qualitative
Grigorovich, A. (2015b). Restricted access: Older lesbian and bisexual women’s experiences with home care services. *Research on Aging*, *37*(7), 763–783.	Age: Range = 55–72; mean = 63.9 Gender: F LGBTQ+ Inclusion: Lesbian and bisexual women Sample Size: 16 Location: Ontario Approach: Qualitative
Grigorovich, A. (2016). The meaning of quality of care in home care settings: Older lesbian and bisexual women’s perspectives. *Scandinavian Journal of Caring Sciences*, *30*(1), 108–116.	Age: Range = 55–72; mean = 64 Gender: F LGBTQ+ Inclusion: Lesbian and bisexual women Sample Size: 16 Location: Ontario Approach: Qualitative
Harbin, A., Beagan, B., & Goldberg, L. (2012). Discomfort, judgment, and health care for queers. *Journal of Bioethical Inquiry*, *9*(2), 149–160, doi:10.1007/s11673-012-9367-x.	Age: Range = 23–73; mean = N/A 60+: 2 Gender: F, Non-binary, Transgender LGBTQ+ Inclusion: Women who identify as lesbian, bisexual, queer or transgender Sample Size: 19 Location: Halifax, Nova Scotia Approach: Qualitative
Hogg, R. S., Craib, K. J., Willoughby, B., Sestak, P., Montaner, J. S., & Schechter, M. T. (1993). Sociodemographic correlates for risk-taking behaviour among HIV seronegative homosexual men. *Canadian journal of public health= Revue canadienne de sante publique*, *84*(6), 423–426.	Age: Risk-takers: Range = 22–47; mean = 35, Controls: Range = 24-66, mean = 40 Gender: M LGBTQ+ Inclusion: Gay men Sample Size: 139 Location: Vancouver, British Columbia Approach: Quantitative
Hoffarth, M., & Bogaert, A. (2017). Opening the closet door: Openness to experience, masculinity, religiosity, and coming out among same-sex attracted men. *Personality and Individual Differences*, *109*, 215–219, doi:10.1016/j.paid.2017.01.011.	Age: Range = 15–78 mean = 36.25 Gender: M LGBTQ+ Inclusion: Same sex attracted men Sample Size: 257 Location: Ontario, Canada Approach: Quantitative
Humble, Á. (2013). Moving from ambivalence to certainty: older same-sex couples marry in Canada. *Canadian Journal on Aging = La Revue Canadienne Du Vieillissement*, *32*(2), 131–144, doi:10.1017/S0714980813000196.	Age: Range = 42–72; mean = 54 Gender: M and F LGBTQ+ Inclusion: Lesbian women and gay and bisexual men Sample Size: 28 Location: Nova Scotia Approach: Qualitative
Katz, A. (2009). Gay and lesbian patients with cancer. *Oncology Nursing Forum*, *36*(2), 203–207, doi:10.1188/09.ONF.203-207.	Age: Range = 39–69; mean = 41.9 65+: 1 Gender: M and F LGBTQ+ Inclusion: Lesbian women and gay men Sample Size: 7 Location: Mid-sized Canadian city Approach: Qualitative
Lacombe-Duncan, A., & Logie, C. (2016). Correlates of clinical breast examination among lesbian, gay, bisexual, and queer women. *Canadian Journal of Public Health*, *107*(4-5), e467–e472, doi:10.17269/CJPH.107.5351.	Age: Range = 18–70; mean = N/A Gender: F, Non-Binary LGBTQ+ Inclusion: Lesbian, queer, bisexual, gay or other women Sample Size: 414 Location: Greater Toronto Area, Ontario Approach: Quantitative
Lee, J. A. (1989). Invisible men: Canada’s aging homosexuals. Can they be assimilated into Canada’s “liberated” gay communities?. *Canadian Journal on Aging/La Revue Canadienne du Vieillissement*,*8*(1), 79–97.	Age: (1) Range = does not specify; (2) Range = 50–80; mean = 60.5 Gender: M LGBTQ+ Inclusion: Older gay men Sample Size: (1) N = does not specify; (2) N = 47 Location: Canada Approach: Qualitative
Lessard, D., Lebouché, B., Engler, K., & Thomas, R. (2016). An analysis of socio-demographic and behavioural factors among immigrant MSM in Montreal from an HIV-testing site sample. *The Canadian Journal of Human Sexuality, 25*(1), 53–60.	Age: Range = 17–75; mean = 37.5 Gender: M LGBTQ+ Inclusion: Men who have sex with men, gay and bisexual males Sample Size: 1353 Location: Montreal, Quebec Approach: Quantitative
Lessard, D., Lebouché, B., Engler, K., Thomas, R., & Machouf, N. (2015). Explaining the appeal for immigrant men who have sex with men of a community-based rapid HIV-testing site in Montreal (Actuel sur Rue). *AIDS Care, 27*(9), 1098–1103.	Age: Range = 18–72; mean = 33 Gender: M LGBTQ+ Inclusion: Men who have sex with men Sample Size: 40 Location: Montreal, Quebec Approach: Qualitative
Liboro, R., & Walsh, R. (2016). Understanding the irony: Canadian gay men living with HIV/AIDS, their Catholic devotion, and greater well-being. *Journal of Religion and Health*, *55*(2), 650–670, doi:10.1007/s10943-015-0087-5.	Age: Range = 31–70; mean = N/A Gender: M LGBTQ+ Inclusion: Gay males Sample Size: 9 Location: Greater Toronto Area, Ontario Approach: Qualitative
Logie, C., & Earnshaw, V. (2015). Adapting and validating a scale to measure sexual stigma among lesbian, bisexual and queer women. *PLoS ONE*, *10*(2), doi:10.1371/journal.pone.0116198.	Age: 18+, Phase 2: Range = 18–70; mean = 31.38, Phase 3: Range = 22–24; mean = 28.69 Gender: F, Non-Binary LGBTQ+ Inclusion: Lesbian, bisexual and/or queer women Sample Size: Phase 1: 10, Phase 2: 466, Phase 3: 44 Location: Toronto, Ontario and Calgary, Alberta Approach: Qualitative
Logie, C., Lacombe-Duncan, A., MacKenzie, R.K. & Poteat, T. (2016). Minority stress and safer sex practices among sexual minority women in Toronto, Canada: Results from a cross-sectional internet-based survey. *LGBT Health 3*(6): 407–415, doi:10.1089/lgbt.2016.	Age: (2) Range =18–70 mean = 31.38 (3) Range =22–44 mean = 28.69 Gender: F LGBTQ+ Inclusion: Lesbian, Bisexual and Queer Women Sample Size: (1) 10 (2) 466 (3) 44 Location: Toronto, Ontario and Calgary, Alberta Approach: Mixed-methods
Low-Beer, S., Bartholomew, K., Weber, A. E., Chan, K., Landolt, M., Oram, D., Schilder, A., & Hogg, R. (2010). A demographic and health profile of gay and bisexual men in a large Canadian urban setting. *AIDS Care, 14*(1), 111–115, doi:10.1080/09540120220097982.	Age: 20+; mean = N/A 65+: 22% of gay and bisexual males Gender: M LGBTQ+ Inclusion: Gay and bisexual males Sample Size: 1176 Location: West-End Vancouver, British Columbia Approach: Qualitative
Lyon, K. A., & Frohard-Dourlent, H. (2015). “Let’s Talk about the Institution”: Same-Sex Common-Law Partners Negotiating Marriage Equality and Relationship Legitimacy. *Canadian Review of Sociology/Revue canadienne de sociologie*, *52*(4), 402–428.	Age: Range = 23–63; mean = 38 60+: 1 Gender: M, F, Non-binary LGBTQ+ Inclusion: Gay men and lesbian, same-sex, queer and unknown women Sample Size: 22 Location: Toronto, Ontario Approach: Qualitative
Macintosh, H., Reissing, E., & Andruff, H. (2010). Same-sex marriage in Canada: The impact of legal marriage on the first cohort of gay and lesbian Canadians to wed. *The Canadian Journal of Human Sexuality*, *19*(3), 79–90.	Age: Range = 23–72; mean = 48.8 Gender: M and F LGBTQ+ Inclusion: Gay men, lesbian women, same-sex couples Sample Size: 52 Location: Ontario and British Columbia Approach: Qualitative
Moody, C., & Smith, N. G. (2013). Suicide protective factors among trans adults. *Archives of Sexual Behavior*,*42*(5), 739–752.	Age: Range = 18–75; mean = 36.75; SD = 13.01 Gender: M, F, Transgender LGBTQ+ Inclusion: Transgender men and women Sample Size: 133 Location: Canada Approach: Qualitative
Morrison, M. A. (2012). Psychological health correlates of perceived discrimination among Canadian gay men and lesbian women. *Canadian Journal of Community Mental Health*, *30*(2), 81–98.	Age: Range = 18–78; mean = 35.65; SD = 12.96 Gender: M and F LGBTQ+ Inclusion: Gay men and lesbian women Sample Size: 348 Location: Canada Approach: Qualitative
Murray, J., & Adam, B. (2001). Aging, sexuality, and HIV issues among older gay men. *The Canadian Journal of Human Sexuality*, *10*(3), 75–90.	Age: Range = 40–79; mean = 45.4 65+: 1 Gender: M LGBTQ+ Inclusion: Gay men Sample Size: 27 Location: Toronto, Ontario Approach: Qualitative
Myers, T., Allman, D., Calzavara, L., Morrison, K., Marchand, R., & Major, C. (1999). Gay and bisexual men’s sexual partnerships and variations in risk behaviour. *Canadian Journal of Human Sexuality, 8*(2), 115–126.	Age: Range = 15–72; mean = 31.9 Gender: M LGBTQ+ Inclusion: Gay and bisexual men Sample Size: ~500 Location: Canada Approach: Quantitative
Myers, T., Godin, G., Lambert, J., Calzavara, L., & Locker, D. (1996). Sexual risk and HIV-testing behaviour by gay and bisexual men in Canada. *AIDS Care*, *8*(3), 297-310.	Age: Range = 16–75; mean = 32.7; SD = 8.8 Gender: M LGBTQ+ Inclusion: Gay and bisexual men Sample Size: 4803 Location: Canada Approach: Qualitative
Myers, T., Orr, K. W., Locker, D., & Jackson, E. A. (1993). Factors affecting gay and bisexual men’s decisions and intentions to seek HIV testing. *American Journal of Public Health, 83*(5), 701–704.	Age: Range = 18–70; mean = 34.3 Gender: M LGBTQ+ Inclusion: Gay and bisexual men Sample Size: 1295 Location: Toronto, Ontario Approach: Quantitative
Myers, T., Tudiver, F. G., Kurtz, R. G., Jackson, E. A., Orr, K. W., Rowe, C. J., & Bullock, S. L. (1992). The Talking Sex Project: descriptions of the study population and correlates of sexual practices at baseline. *Canadian journal of public health= Revue canadienne de sante, 83*(1), 47–52.	Age: Range = 14–72; mean = 32 Gender: M LGBTQ+ Inclusion: Gay and bisexual men Sample Size: 612 Location: Toronto, Ontario Approach: Quantitative
O’Neill, T. J., Raboud, J. M., Tinmouth, J., Rourke, S. B., Gardner, S., Cooper, C., ... & OHTN Cohort Study Team. (2017). Burden and risk factors for gastrointestinal symptom distress in HIV patients in the modern antiretroviral era. *AIDS care*, *29*(2), 156–167.	Age: 16+; mean = 45.7 Gender: M LGBTQ+ Inclusion: Men who have sex with men Sample Size: 1532 Location: Ontario Approach: Qualitative
Persson, T., Pfaus, J., & Ryder, A. (2015). Explaining mental health disparities for non-monosexual women: Abuse history and risky sex, or the burdens of non-disclosure? *Social Science & Medicine*, *128*, 334–335, doi:10.1016/j.socscimed.2014.08.038.	Age: Range = 18–66; mean = 24.4 Gender: F LGBTQ+ Inclusion: Bisexual, mostly lesbian women Sample Size: 388 Location: National
Ploeg, J., Lohfeld, L., & Walsh, C. (2013). What is “elder abuse”? Voices from the margin: The views of underrepresented Canadian older adults. *Journal of Elder Abuse & Neglect*, *25*(5), 396–424, doi:10.1080/08946566.2013.780956.	Age: 60+; mean = N/A Gender: F LGBTQ+ Inclusion: Lesbian women Sample Size: 87 Location: Canada Approach: Qualitative
Rank, C., Gilbert, M., Ogilvie, G., Jayaraman, G. C., Marchand, R., Trussler, T., Hogg, R. S., Gustafson, R., Wong, T., & The ManCount Study Team (2012). Acceptability of human papillomavirus and sexual experience prior to disclosure to health care providers among men who have sex with men in Vancouver, Canada: Implications for targeted vaccination programs. *Vaccine, 30*, 5755–5760	Age: Range = 19–83; mean = N/A Gender: M LGBTQ+ Inclusion: Men who have sex with men, gay and bisexual men Sample Size: 1169 Location: Vancouver, British Columbia
Remis, R., Alary, M., Otis, J., & Hogg, R. (2000). HIV infection and risk behaviors in young gay and bisexual men / the authors respond. *Canadian Medical Association. Journal*, *163*(1), 14.	Age: Range = 18–73; mean = 34.5 Gender: M LGBTQ+ Inclusion: Men who have sex with men Sample Size: 650 Location: Montreal, Quebec Approach: Quantitative
Remis, R., Liu, J., Loutfy, M., Tharao, W., Rebbapragada, A., Huibner, S., Kesler, M., Halpenny, R., Grennan, T., Brunetta, J., Smith, G., Reko, T., & Kaul, R. (2016). Prevalence of sexually transmitted viral and bacterial infections in HIV-positive and HIV-negative men who have sex with men in Toronto. *PloS One*, *11*(7), e0158090, doi:10.1371/journal.pone.0158090.	Age: Range=22+ Mean = 44.35 Gender: M LGBTQ+ Inclusion: Gay men and other men who have sex with men Sample Size: 442 Location: Toronto, Ontario Approach: Quantitative
Ross, L. E., Bauer, G. R., MacLeod, M. A., Robinson, M., MacKay, J., & Dobinson, C. (2014). Mental health and substance use among bisexual youth and non-youth in Ontario, Canada. *PLoS one*, *9*(8), e101604.	Age: 16+; mean = N/A 55+: 12 Gender: M and F LGBTQ+ Inclusion: Bisexual males and females Sample Size: 405 Location: Ontario Approach: Quantitative
Ross, L. E., O’Gorman, L., MacLeod, M. A., Bauer, G. R., MacKay, J., & Robinson, M. (2016). Bisexuality, poverty and mental health: A mixed methods analysis. *Social Science & Medicine, 156*, 64–72.	Age: Range = 25–66; mean = N/A 55+: 12 Gender: M, F, Non-Binary, Transgender LGBTQ+ Inclusion: Bisexual men and women, genderqueer, 2-spirited, trans men and women, and other Sample Size: 302 Location: Ontario Approach: Mixed-methods
Rotondi, N. K., Bauer, G. R., Scanlon, K., Kaay, M., Travers, R., & Travers, A. (2013). Nonprescribed hormone use and self-performed surgeries: “Do-it-yourself” transitions in transgender communities in Ontario, Canada. *American Journal of Public Health*, *103*(10), 1830–1836.	Age: 16+ 65+: 2.5% Gender: M, F, Transgender LGBTQ+ Inclusion: Transgender men and women Sample Size: 433 Location: Ontario Approach: Qualitative
Scheim, A. I., & Bauer, G. R. (2015). Sex and gender diversity among transgender persons in Ontario, Canada: results from a respondent-driven sampling survey. *The Journal of Sex Research*, *52*(1), 1–14.	Age: Range = 16–77; mean = N/A Gender: M, F, Non-Binary, Transgender LGBTQ+ Inclusion: Transgender males, females and fluid Sample Size: 433 Location: Ontario Approach: Quantitative
Scheim, A., Bauer, G., & Shokoohi, M. (2016). Heavy episodic drinking among transgender persons: Disparities and predictors. *Drug and Alcohol Dependence*, *167*, 156–162, doi:10.1016/j.drugalcdep.2016.08.011.	Age: 16+; mean = N/A Gender: M, F, Non-Binary, Transgender, Transsexual LGBTQ+ Inclusion: Transgender, transsexual, or transitioned including genderqueer or other non-binary gender identities Sample Size: 433 Location: Ontario Approach: Quantitative
Shuper, P. A., & Fisher, W. A. (2008). The role of sexual arousal and sexual partner characteristics in HIV+ MSM’s intentions to engage in unprotected sexual intercourse. *Health Psychology*, *27*(4), 445.	Age: Range = 26–66; mean = 44 Gender: M LGBTQ+ Inclusion: Men who have sex with men Sample Size: 77 Location: Ontario Approach: Quantitative
Sinding, C., Grassau, P., & Barnoff, L. (2007). Community support, community values: The experiences of lesbians diagnosed with cancer. *Women & Health*, *44*(2), 59–79.	Age: Range = 36–72; mean = 50 Gender: F LGBTQ+ Inclusion: Lesbian women Sample Size: 26 Location: Ontario Approach: Quantitative
Sinding, C., Barnoff, L., & Grassau, P. (2004). Homophobia and heterosexism in cancer care: The experiences of lesbians. *CJNR (Canadian Journal of Nursing Research)*, *36*(4), 170–188.	Age: Range = 36–72; mean = 50 Gender: F LGBTQ+ Inclusion: Lesbian women Sample Size: 26 Location: Ontario Approach: Qualitative
Soskolne, C., Coates, R., & Sears, A (1986). Characteristics of a male homosexual/bisexual study population in Toronto, Canada. *Canadian Journal of Public Health = Revue Canadienne de Sante Publique*, *77*(1), 12–16, doi:10.1007/s10508-009-9556-9.	Age: Range = 17–67; mean = 30.8 Gender: M LGBTQ+ Inclusion: Men who have sex with men, gay and bisexual men Sample Size: 339 Location: Toronto, Ontario Approach: Quantitative
Wallach, I., & Brotman, S. (2013). Ageing with HIV/AIDS: A scoping study among people aged 50 and over living in Quebec. *Ageing & Society*, *33*(7), 1212–1242, doi:10.1017/S0144686X12000529.	Age: Range = 50–68; mean = N/A Gender: M and F LGBTQ+ Inclusion: Gay men and women Sample Size: 9 Location: Quebec Approach: Qualitative
Yuan, L., & Robinson, G. (1994). Hepatitis B vaccination and screening for markers at a sexually transmitted disease clinic for men. *Canadian Journal of Public Health = Revue Canadienne de Sante Publique*, *85*(5), 338–341.	Age: Gay/Bisexual Men Range = 16–68; mean = 30.3, Heterosexual Men Range = 16–51; mean = 27.4 Gender: M LGBTQ+ Inclusion: Gay and bisexual men Sample Size: 887 Location: Toronto, Ontario Approach: Quantitative
